# Pleuropulmonary blastoma, a rare entity in childhood

**DOI:** 10.1259/bjrcr.20200206

**Published:** 2021-04-29

**Authors:** Pihou Gbande, Tawfiq Abukeshek, Fouad Bensari, Safa El-Kamel

**Affiliations:** 1 André Grégoire Intercommunal Hospital Center, Medical Imaging Service, Montreuil, France; 2 Department of Radiology, University Hospital Center Campus, Lome, Togo; 3 Department of Radiology, Al-Makassed Hospital, Ruba el-Adawiya St, Jerusalem, Palestine

## Abstract

Pleuropulmonary blastoma (PPB) is a rare malignant intrathoracic mesenchymal tumour with a variable aggressiveness. It is the most common primary malignancy in the lung during childhood. In this study, we present a case of a 3-year-old male child who complained of persistent dry cough. Radiographs suggested the diagnosis of PPB, which was been confirmed by the histo-pathological examination of a biopsy taken from the tumour under CT guidance. This case was reported to emphasise the importance of radiology, whether diagnotic or interventional, in diagnosing rare cases such as PPB.

## Introduction

Pleuropulmonary blastoma (PPB) accounts for 0.25 to 0.5% of all primary lung neoplasms.^1^ They are malignant tumours that can be pleural, pulmonary or both. Despite being rare, they are considered the most common primary cancers in the lung during childhood,^2^ especially in those who are under 5 years of age.^3^ The first two years of age remain the highest risk for developing PPB, with about 90% of the cases occuring within this age group.^3^ PPB is divided into type I, II & III according to histological type of the tumour. Each of the subtypes correlates to the age at diagnosis and the overall prognosis of the patient.^4^ The main aim of reporting this case is to highlight the importance of both diagnostic and interventional radiology in helping other physicians to reach rare diagnosis correctly.

## Case presentation

A 3-year-old male child presented to our emergency department with a history of persistent non-productive cough of 4 days duration. He had a history of hospitalisation once for acute bronchiolitis at the age of 1 year. His family history was unremarkable. Upon clinical evaluation, he had tachypnea, intercostal retractions, absent air entry on the right lung and abdominal distention. Otherwise, the clinical examination was normal. Laboratory investigations revealed leukocytosis up to 14,900 / mm^3^, an elevated C reactive protein (CRP) level of 108 mg/l, and anaemia with a haemoglobin level of 9.8 g dl^−1^.

**Figure 1.**
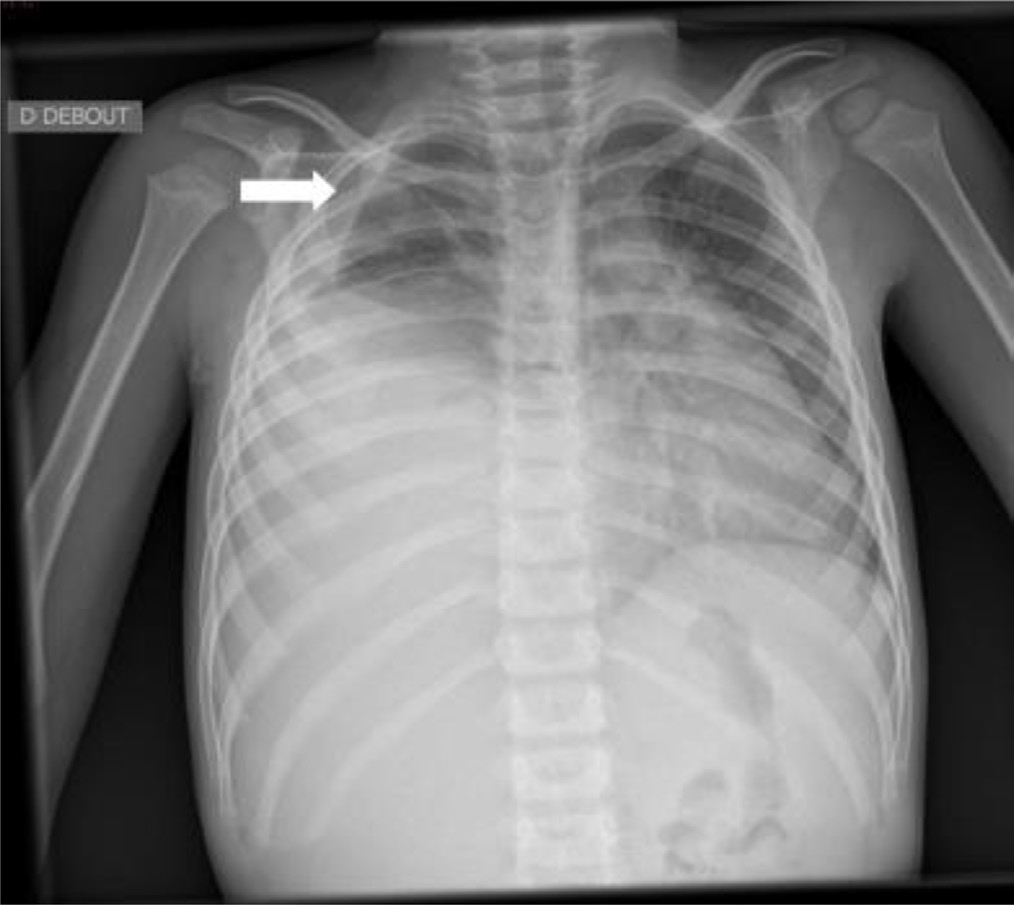
Chest X-ray showing a large, rounded homogeneous opacity in the right mid and lower zones with shifting of the mediastinum to the opposite side, mild pleural effusion (white arrow) in the right upper zone.

**Figure 2.**
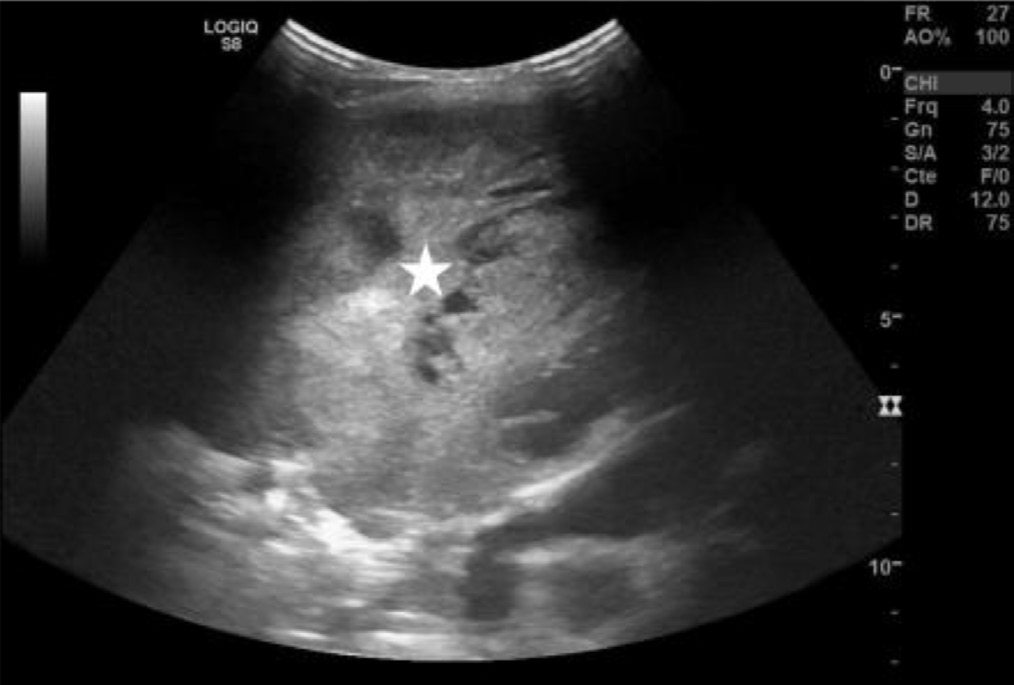
Ultrasonography image showing a large heterogeneous echogenic mass (white star) at base of right pleura.

**Figure 3.**
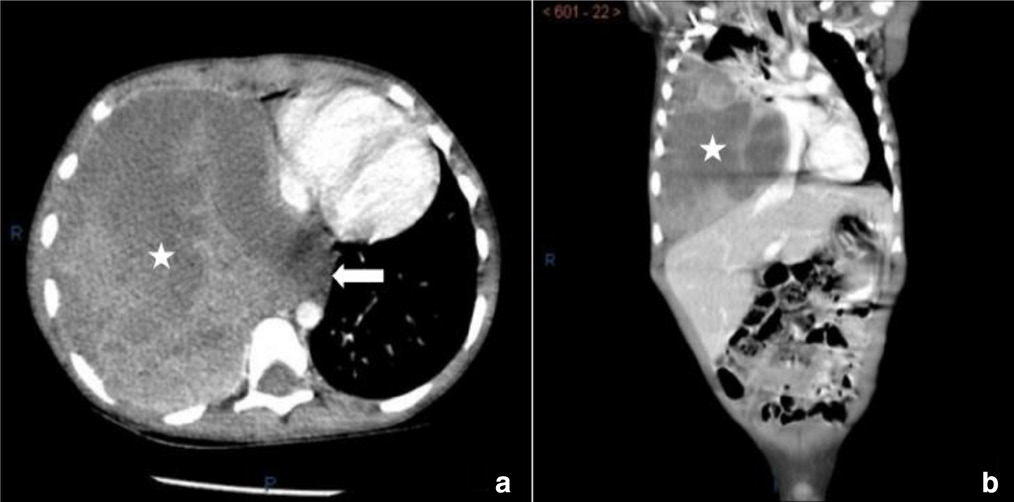
Contrast-enhanced axial image (**A**) and coronal image (**B**) of CT chest in the mediastinal window. We notice a large well-circumscribed mass of heterogeneous attenuation in the region of the lower lobe of the right lung (white stars). Note extension of mass across midline (arrow).

A chest X-ray (Figure 1) was performed and showed a large rounded homogenous opacity occupying the right middle and lower zones, with shifting of the mediastinum to the opposite side, alongside a mild (loculated) pleural effusion within the right upper zone. The radiograph showed no evidence of calcifications, cavitations, air bronchogram or underlying bony destruction. Chest ultrasound (Figure 2) was done and showed a large heterogeneous echogenic mass at the base of right pleural cavity with a mild pleural effusion within the upper lobe. No liver invasion was appreciated. CT scan with IV contrast (Figure 3) was done for further assement and revealed a well-defined heterogeneous soft tissue density lesion measuring 11 × 8 × 14 cm (AP × TR × CC) in the right lower lobe. The lesion showed enhancing areas and some non-enhancing areas, upon contrast administration, suggesting a presence of necrosis, in addition to a mass effect shifting the mediastinum to the opposite side and resulting in passive atelectasis of the right upper lobe associated with a small right upper lobe pleural effusion. No evidence of calcification within the lesion or chest wall invasion could be seen. There is no associated cystic lung disease in remaining lungs. No evidence of metastasis in chest or abdomen could be appreciated. A CT-guided biopsy was taken from the tumour. Histo-pathological analysis of the biopsy revealed a PPB type III. Therefore, the patient was referred for chemotherapy.

## Discussion

Lung malignant tumours during childhood are considered a rare entity, representing only 0.5–1% of primary lung cancers. Pulmonary blastomas are uncommon fast-growing lung neoplasms that account for approximately 0.25–0.5% of primary lung malignancies.^1^ The pathogenesis of these tumours remains uncertain, but due to their resemblance to fetal lung tissue, pluripotent pulmonary blastomas have been postulated to be the origin of the disease. They occur mostly in the infants and young children, however, similiar cases had been reported in adults.^5–7^

Up to date, no definite causative agent has been determined, but some associations have been reported. PPBs are associated with type IV congenital pulmonary airway malformations.^8^ They are also associated with dysplasia syndrome in 33% of the cases.^9^ The association of DICER1 mutations and PPB is reported in approximately 66% of the cases.^2^ Therefore, all patients with PPB should be screened for DICER1 mutation. If DICER1 mutation is detected during screening, the possibility for the patient to develop malignancies in other organs must be considered. In 30% of cases, patients with PPB may also present with multilocular cystic nephroma, and, very rarely, Wilms tumor.^9^ Following the initial description by Barrett and Barnard in 1945 (originally termed embryoma of the lung), pulmonary blastomas were classified into three histological subtypes^10^ : I, II, and III. Type I is a purely cystic lesion (14%), type II tumours have both cystic and solid components (48%) and type III are purely solid (38%). The classification has also a prognostic value, type I being the least malignant and type III the most malignant lesion.

The clinical picture of blastomas is highly variable. There are no specific presenting signs or symptoms of biphasic pulmonary blastomas. Some studies suggest that symptoms present only in 60% of the cases, with the lesions for the rest of the cases found incidentally.^10,11^ Symptoms, when present, may include fever, dyspnoea, respiratory distress, cough. Rarely, hemoptysis, chest pain, weight loss, anorexia, fatigue, or even neurologic symptoms can occur. Currently, there is no specific serum marker for pulmonary blastomas.^10^

Imaging studies have enormous capability on identifying the charactersticis of lung lesions. Usually, PPB appears as a solitary, well-demarcated, peripheral mass that can be large enough to extend beyond 10 cm in size.^12^ PPB is often found late on plain film radiographs as a unilateral lung whiteout with a mediastinal shift to the opposite side. Typically, there are no adjacent rib erosions or calcifications. In the majority of cases, the disease is unilateral, but bilateral disease has been reported. In some cases, pleural effusion may be seen.^11^ Ultrasound is a poor imaging modality for diagnosing PPB as findings are non-specific and may show a large region of consolidation without sonographic air bronchogram. The appearance of PPB on CT scan is variable depending on the histological type of the tumour.^8,13,14^ Type I lesions manifest as a single cyst or a multicystic lesion (often air-filled). Type II lesions show air- or fluid-filled cavities with possible air-fluid levels along with solid internal nodules. In type III lesions, neoplasms are solid and show low attenuation, with homogeneous or heterogeneous enhancement. On MRI, the tumor manifests as a heterogeneous mass, typically hyperintense on both T1W and T2W sequences.^15^ A FDG-PET CT scans reveals a hypermetabolic lesion. PET CT is helpful in evaluating metastatic work up. It is also helpful in evaluating tumour vitality and judging the response to neoadjuvant chemotherapy.^16^

The differential diagnosis for a chest wall mass identified on imaging of a young child is wide. For type 1 PPB lesions, the differential diagnosis includes other cystic lesions such as bronchogenic cyst, lung cyst, pneumatocele, and pulmonary interstitial emphysema. The differential diagnosis for types II and III PPBs, particularly when they are locally aggressive, includes more common tumours such as neuroblastoma, Ewing’s sarcoma, Askin tumour and rhabdomyosarcoma. However, chest lesions that arise from the chest wall such as Ewing’s sarcoma and Askin tumour usually demonstrate findings of chest wall involvement. Neuroblastoma typically arises in the posterior mediastinum and demonstrates chest wall involvement as rib erosion. Rhabdomyosarcoma can also involve the chest wall.

Identifying imaging findings that can make a specific diagnosis more or less likely. Interpetation of imaging exams was important and helped in making the correct diagnosis in this case by identifying the heterogeneousity, low attenuation, the association of pleural effusion and the absence of chest wall invasion. The definitive diagnosis of PPB was made by cytogenic analysis of the tumor cells, which were biopsied under the guidance of CT.

## Learning points

- Despite being rare, PPB affects mainly young children and can lead to quick clinical detrioration.
- When a large hemithorax mass is identified in a young child, PPB should be considered in the differential diagnosis.
- The imaging findings favouring the possibility of PPB on a chest radiograph include large mass lesion with small pleural effusion, contralateral mediastinal shift and lack of chest wall invasion.
